# Multi-Level Biological Network Analysis and Drug Repurposing Based on Leukocyte Transcriptomics in Severe COVID-19: In Silico Systems Biology to Precision Medicine

**DOI:** 10.3390/jpm12071030

**Published:** 2022-06-23

**Authors:** Pakorn Sagulkoo, Hathaichanok Chuntakaruk, Thanyada Rungrotmongkol, Apichat Suratanee, Kitiporn Plaimas

**Affiliations:** 1 Program in Bioinformatics and Computational Biology, Graduate School, Chulalongkorn University, Bangkok 10330, Thailand; pakorn.sagulkoo@cmu.ac.th (P.S.); hathaichanok.chuntakaruk@gmail.com (H.C.); t.rungrotmongkol@gmail.com (T.R.); 2 Center of Biomedical Informatics, Department of Family Medicine, Faculty of Medicine, Chiang Mai University, Chiang Mai 50200, Thailand; 3 Center of Excellence in Biocatalyst and Sustainable Biotechnology Research Unit, Department of Biochemistry, Faculty of Science, Chulalongkorn University, Bangkok 10330, Thailand; 4 Department of Mathematics, Faculty of Applied Science, King Mongkut’s University of Technology North Bangkok, Bangkok 10800, Thailand; apichat.s@sci.kmutnb.ac.th; 5 Intelligent and Nonlinear Dynamics Innovations Research Center, Science and Technology Research Institute, King Mongkut’s University of Technology North Bangkok, Bangkok 10800, Thailand; 6 Advance Virtual and Intelligent Computing (AVIC) Center, Department of Mathematics and Computer Science, Faculty of Science, Chulalongkorn University, Bangkok 10330, Thailand; 7 Omics Science and Bioinformatics Center, Faculty of Science, Chulalongkorn University, Bangkok 10330, Thailand

**Keywords:** severe COVID-19, systems biology, key genes, novel biomarkers, drug repurposing

## Abstract

The coronavirus disease 2019 (COVID-19) pandemic causes many morbidity and mortality cases. Despite several developed vaccines and antiviral therapies, some patients experience severe conditions that need intensive care units (ICU); therefore, precision medicine is necessary to predict and treat these patients using novel biomarkers and targeted drugs. In this study, we proposed a multi-level biological network analysis framework to identify key genes via protein–protein interaction (PPI) network analysis as well as survival analysis based on differentially expressed genes (DEGs) in leukocyte transcriptomic profiles, discover novel biomarkers using microRNAs (miRNA) from regulatory network analysis, and provide candidate drugs targeting the key genes using drug–gene interaction network and structural analysis. The results show that upregulated DEGs were mainly enriched in cell division, cell cycle, and innate immune signaling pathways. Downregulated DEGs were primarily concentrated in the cellular response to stress, lysosome, glycosaminoglycan catabolic process, and mature B cell differentiation. Regulatory network analysis revealed that hsa-miR-6792-5p, hsa-let-7b-5p, hsa-miR-34a-5p, hsa-miR-92a-3p, and hsa-miR-146a-5p were predicted biomarkers. *CDC25A*, *GUSB*, *MYBL2*, and *SDAD1* were identified as key genes in severe COVID-19. In addition, drug repurposing from drug–gene and drug–protein database searching and molecular docking showed that camptothecin and doxorubicin were candidate drugs interacting with the key genes. In conclusion, multi-level systems biology analysis plays an important role in precision medicine by finding novel biomarkers and targeted drugs based on key gene identification.

## 1. Introduction

Nowadays, our world has experienced the coronavirus disease 2019 (COVID-19) pandemic, causing numerous morbid and mortal cases. The disease is caused by severe infection of acute respiratory syndrome coronavirus-2 (SARS-CoV-2). The virus is a positive-sense single-strand RNA β-coronavirus classified in the Coronaviridae family, which also consists of SARS-CoV and middle east respiratory syndrome coronavirus (MERS-CoV) [1]. These viruses all emerged within the first 20 years of the 21st century and caused numerous public health and economic issues. Comparative genomics studies have revealed that the SARS-CoV-2 genome resembles the SARS-CoV sequence, with 79% identity. In contrast, the MERS-CoV sequence shares only 50% identity with SARS-CoV-2’s sequence [2]. Moreover, phylogenetic analysis using whole-genome sequences and phylogenetic tree construction by the neighbor-joining method reveals that SARS-CoV-2 is clustered in the sarbecovirus group and the virus is close to coronaviruses in bats and pangolins [2].

For the global statistics, the number of confirmed cases and deaths of COVID-19 from World Health Organization (WHO) data on 21 June 2022 were 537,591,764 and 6,319,395, respectively [3]. In addition, the global fatality rate is 3.4%. The rate is higher than seasonal flu but lower than SARS-CoV and MERS-CoV infections [4,5]. Despite the assumption that bats were hosts in this zoonotic infection, several studies have indicated that the disease occurred via an intermediate host such as pangolins [6,7]. The disease’s main transmission route is receiving infectious respiratory droplets from direct person-to-person contact [8,9]. SARS-CoV-2 can spread in all stages of the disease: asymptomatic, presymptomatic, and symptomatic stages [6]. The median incubation period is approximately 5.1 days, and most people (97.5%) have symptoms within 11.5 days. Only 1% of patients develop symptoms after 14 days of quarantine [10]. The most common clinical features of COVID-19 are dry cough, fever, fatigue, and myalgia. Some patients have gastrointestinal symptoms, for instance, nausea, anorexia, and diarrhea [11,12,13]. Less common clinical presentations include sputum production, headache, and hemoptysis [11,12]. About 64% to 80% of patients present with anosmia or ageusia [14,15,16]. Furthermore, at least 50% of patients will progress to dyspnea [17]. Progressive dyspnea and hypoxemia usually develop approximately one week after the clinical onset [18]. Acute respiratory distress syndrome (ARDS), characterized by severe hypoxemia, and bilateral pulmonary edema that cannot be explained by cardiac causes or volume overload, is a condition mainly found in severe COVID-19 [18]. Several risk factors contributing to severe illness include older age, chronic lung diseases, cardiovascular diseases, diabetes mellitus, obesity, chronic kidney diseases, immunocompromised host, and cancers [12,18]. Nearly 17% to 35% of admitted patients needed intensive care units (ICU) due to respiratory failure. Approximately 29% to 91% of patients in ICU obtain mechanical ventilation [19,20,21,22]. The main causes of death are ARDS, acute respiratory failure, coagulopathy, septic shock, metabolic acidosis, cardiovascular complications, and multiple organ failure [23].

Pathogenesis and pathophysiology of COVID-19 are required for further studies. The disease is classified into two stages: early and late stages [9]. In the early stage, SARS-CoV-2 infects host cells and initiates proliferation. It enters respiratory epithelial cells and alveolar cells via using spike (S) protein, primed by host transmembrane serine protease *2* (TMPRSS2), binding to host membrane receptors, for example, angiotensin-converting enzyme 2 (ACE2) [24,25]. While viral replication occurs, the immune system will proceed. Hence, mild constitutional symptoms arise in this stage. The innate immunity will recruit myeloid-lineage leukocytes such as macrophages, neutrophils, and natural killer (NK) cells to alveolar tissue [8]. In the late stage, pulmonary tissue damage and hyperinflammation emerge from excessive proinflammatory cytokine secreted from these leukocytes. Pneumocytes and alveolar endothelial cells are injured and dead, resulting in interstitial fluid leakage; therefore, pulmonary edema will occur and progress to ARDS later [24]. Accumulation of fluid in alveolar space and pneumocyte damage leads to impaired gas exchange, causing hypoxia and hypercapnia [24]. Furthermore, some patients will develop to hyperinflammation stage or cytokine storm caused by excessive proinflammatory cytokines such as interferon α (IFN-α), IFN- β, IFN-γ, interleukin 1β (IL-1β), IL-6, IL-12, IL-18, IL-33, and tumor necrosis factor α (TNF-α) [26]. Cytokine storm is characterized by cytokine overproduction causing collateral tissue damage [27]. Uncontrolled cytokine storms can lead to multiple organ dysfunction and failure in the last stage [27]. Severe COVID-19 cases usually die due to cytokine storms with multiple organ failures [28]. 

The gold standard diagnostic testing of COVID-19 is the reverse transcriptase-polymerase chain reaction (RT-PCR) from nasal and throat swab samples [29]. The specificity of PCR is nearly 100% if there are no contaminations. Antigen tests have benefits over PCR as they have lower costs and are used in the point-of-care setting, though they have sensitivity less than PCR [30,31]. Nevertheless, there are still no effective diagnostic testing or biomarkers used to predict the possibility of severe illness progression precisely. The primary treatment for COVID-19 is the best supportive care and respiratory support [23,24]. Medical therapies include anti-inflammatory agents using corticosteroids and antiviral treatments such as ritonavir and favipiravir [32,33,34]. In addition, the role of vaccines in COVID-19 prevention has been studied and needs further investigation. Although the current treatments improve the disease, they cannot cover all patients with severe conditions. As a result, discovering novel biomarkers and targeted drugs based on cytokine storm and impaired immune-associated key genes and proteins could play a crucial role in predicting and improving COVID-19 severity. 

In the bioinformatics and precision medicine era, systems biology and multi-omics studies allow translational medicine to be effective in clinical practices [35]. Several combined wet and dry experimental studies have provided invaluable information in molecular biology and medicine [36,37,38]. Moreover, a combination of knowledge between biology, computer science, statistics, and mathematics explores the underlying molecular mechanisms of numerous diseases such as cancer, degenerative diseases, genetic diseases, etc. Structural information on protein-related SARS-CoV-2 such as S protein, main protease (M^pro^), and papain-like protease (PL^pro^), obtained from the Protein Data Bank (PDB), has also provided the details on physical protein interactions and benefits for identifying drug–protein interaction in COVID-19 via protein binding site analyses [39,40,41,42,43,44,45]. One of the most powerful tools used in bioinformatics is network analysis. With the use of network analysis, central node identification using various centrality measurements and community detection by several network clustering algorithms [46,47] have been widely used in much research. These approaches were successfully applied in several applications to identify key disease-related genes, disease–disease associations, disease–protein associations, and drug–disease associations [48,49,50,51,52,53,54,55,56,57]. Additionally, the benefit of the network analysis is drug repositioning or drug repurposing, characterized by discovering a new role of treatment from existing drugs based on the key disease-related genes identified from the biological network [58]. Structural bioinformatics also plays a vital role in drug repurposing via finding physical interactions between targeted proteins from PDB structures and drugs using molecular docking [59,60,61]. In addition, novel biomarkers can be recognized from the network analysis [62].

In this study, we proposed multi-level biological networks analysis, such as regulatory and protein–protein interaction (PPI) network, based on leukocyte transcriptomic profiles to identify novel biomarkers and key genes in severe COVID-19. Furthermore, drug repurposing was performed based on drug–gene and drug–protein interaction database searching and molecular docking. This study aims to discover novel biomarkers and candidate targeted drugs to predict and treat severe COVID-19 at clinical levels by applying various biological data and networks.

## 2. Materials and Methods

**Figure 1:**
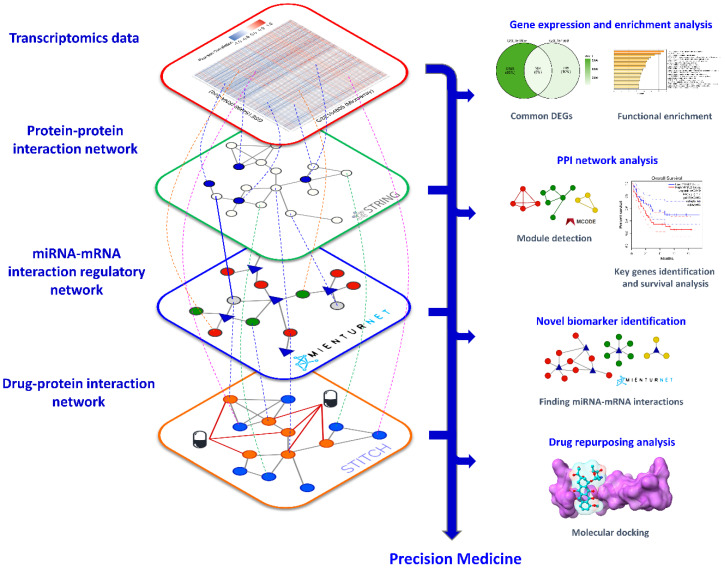
Diagram summarizes the process of identifying key genes, novel biomarkers, and candidate drugs using multi-levels of biological network analyses. There are four principal data and networks, including transcriptomics data, protein–protein interaction network, miRNA–mRNA interaction regulatory network, and drug–protein interaction network towards precision medicine.

The overall process of identifying key genes, novel biomarkers, and candidate drugs using several levels of the biological network is summarized in Figure 1. All our proposed methods were dry experiments or in silico studies based on wet experimental data acquisition from databases. First, the leukocyte transcriptomic profiles from Gene Expression Omnibus (GEO) datasets [50] were downloaded to indicate an overall immune status in severe COVID-19 patients compared to controls. Common differentially expressed genes (DEGs) were identified by considering statistical criteria described in Section 2.1 Data Collection and Preprocessing. The functional enrichment analysis of upregulated and downregulated DEGs were conducted using Metascape [63]. Second, STRING v11.0 [64] was used to construct the PPI network based on the common DEGs. Network clustering was conducted using the Molecular Complex Detection (MCODE) plugin in Cytoscape [65]. The degree and betweenness centrality were calculated using Network Analyzer in Cytoscape to find hub and bottleneck genes in the PPI network. Additionally, the survival analysis from Gene Expression Profiling Interactive Analysis (GEPIA2) [66], using acute myeloid leukemia (LAML) as a cell type model, was operated to identify key genes from the hub and bottleneck genes. Third, MicroRNA Enrichment Turned Network (MIENTURNET) [67,68] was used to construct regulatory networks and identify novel biomarkers. Finally, drugs resulting from drug–gene and drug–protein interaction databases were studied by molecular docking.

### 2.1. Data Collection and Preprocessing

Two gene expression datasets (GSE164805 and GSE154998) were downloaded from GEO DataSets (https://www.ncbi.nlm.nih.gov/geo/, accessed on 14 January 2022) [69]. Both datasets are leukocyte transcriptomic profiles collected from peripheral blood samples in severe COVID-19 patients compared to non-COVID-19 controls. The gene expression method in GSE164805 was conducted based on the microarray technique, while GSE154998 measured the transcriptomic profiles via the RNA sequencing (RNA-Seq) method [70,71]. The complete data sets, consisting of false discovery rate (FDR) *q*-value and log2fold change (log2 FC), were manipulated using R package ‘dplyr’ [72]. The DEGs were filtered based on genes expression having the FDR < 0.05 and absolute log2 FC (|log2 FC|) > 1. DEGs that met the criteria in both data sets were common DEGs that were used for further analysis. Moreover, common DEGs with log2 FC > 1 and log2 FC < −1 were considered upregulated and downregulated DEGs, respectively.

### 2.2. Functional Enrichment Analysis Based on Up- and Downregulated DEGs

Metascape (https://metascape.org/gp/index.html#/main/step1, accessed on 20 January 2022) [63] was performed for functional enrichment analysis of the upregulated and downregulated DEGs. Metascape is a web-based portal integrating functional enrichment, interactome analysis, gene annotation, and membership search from over 40 knowledgebases [63]. Functional and pathway terms used in the software include Gene ontology biological process (GO-BP) [73], Kyoto Encyclopedia of Genes and Genomes (KEGG) pathways [74], Reactome pathways [75], WikiPathways [76], Canonical [77], and CORUM pathway [78]. The functional enrichment analysis in the software was performed using a hypergeometric test and Benjamini–Hochberg statistical correction algorithm [63]. Enrichment terms with a significant level (FDR *q*-value < 0.01) were selected.

### 2.3. PPI Network Construction from the Common DEGs

STRING v11.0 (https://string-db.org/, accessed on 20 January 2022) [64], a protein interactome online database collecting a human interactome consisting of 19,556 proteins and 11,938,498 interactions, was performed to construct the PPI network without adjacent node expansion using the common DEGs as the input. The PPI network was built with an interaction confidence score greater than 0.400 (medium confidence). The confidence score of the interaction is the probability value calculated based on both experimental and computational evidence such as text mining, high-throughput experiments, co-expression and gene fusion data, and information from other databases. Furthermore, the PPI network was downloaded and exported to Cytoscape 3.9.0 (https://cytoscape.org/ accessed on 20 January 2022) [65], a biological network visualizing software.

### 2.4. Topological and Network Clustering Analysis of the PPI Network

In Cytoscape, the Network Analyzer plugin was performed to calculate global topological parameters, for instance, average degree, diameter, radius, average clustering coefficient, average shortest path length, and network density. Local topological parameters, such as degree, closeness, betweenness, and clustering coefficient, were also computed. Moreover, network clustering was conducted using MCODE plugin [79] in Cytoscape. The plugin was used by default settings, for example, a degree cut-off: 2, node score cut-off: 0.2, *k*-core: 2, and max depth: 100. An MCODE score cut-off for cluster selection was greater than 5.

### 2.5. Regulatory Network Construction and Novel Biomarkers Identification

The gene sets in each MCODE cluster were inputted in MIENTURNET (http://userver.bio.uniroma1.it/apps/mienturnet/, accessed on 22 January 2022) [67], an online-based software, to construct microRNA (miRNA)–mRNA regulatory networks. The software was used to find miRNA–mRNA interactions based on miRTarBase, a miRNA-target database validated from experimental data [68]. miRNAs with interaction FDR *q*-value less than 0.05 were considered novel biomarkers in severe COVID-19.

### 2.6. Identification of Hub and Bottleneck Genes

Degree and betweenness centrality were measured using the Network Analyzer plugin in the Cytoscape to find the hub and bottleneck genes in the PPI network. Given a network called *G*, let *A* be a non-weight adjacency matrix of network *G*. Degree centrality ($C_{D}$) is the number of adjacent nodes interacting with interested node *i*, according to this equation

(1) $$C_{D}\left(i\right)=\underset{j}{\sum }A_{ij}\;,\;$$

where *A_ij_* is a value of matrix *A* of node *i* and *j*, respectively. In biological networks, the high-degree nodes are hub genes playing a crucial role in the network function due to numerous interactions. Nodes in the PPI network having degree centrality greater than the 95th percentile were considered hub genes.

Betweenness centrality ($C_{B}$) is the summation of the ratio between the shortest path of node *u* and *v* that pass through node *i*. The betweenness centrality is calculated based on this equation

(2) $$C_{B}\left(i\right)=\underset{u\;\neq \;v\;\neq \;i}{\sum }\frac{\sigma_{uv}\left(i\right)}{\sigma_{uv}}\;,$$

where $\sigma_{uv}$ is a total number of the shortest path between node *u* and *v* and $\sigma_{uv}\left(i\right)$ is the number of the shortest path between node *u* and *v* that pass through node *i*. Nodes with betweenness more than 95th percentile were bottleneck genes in the PPI network. The bottleneck nodes play an important function in forming the bridges controlling the flow of information in the network.

### 2.7. Finding Key Genes Using Survival Analysis

Because there is no powerful tool to validate and predict the gene essentiality in severe COVID-19 recently, we applied the cancer survival analysis to find key genes from the PPI network. The key genes in severe COVID-19 were identified based on the hub and bottleneck genes by using GEPIA2 (http://gepia2.cancer-pku.cn/#index, accessed on 25 January 2022) [66]. GEPIA2 provides the single gene essentiality in several cancer types by using survival and gene expression analysis based on The Cancer Genome Atlas (TCGA) [80] and Genotype-Tissue Expression (GTEx) data [81]. As earlier described, the myeloid-lineage leukocytes such as macrophages, neutrophils, and NK cells play a vital role in COVID-19-associated cytokine storm by releasing the excessive proinflammatory cytokines. Hence, LAML was used as a cell type model to find the key genes related to immune-induced severe COVID-19. The survival analysis was performed by the Kaplan–Meier method, which considers these parameters such as log-rank *p*-value and hazard ratio (HR) with 95% confidence interval.

### 2.8. Drug Repurposing Based on the Key Genes

#### 2.8.1. Drug–Gene and Drug–Protein Interaction Database Searching

The key genes were inputted to find targeted drugs in these drug–gene interaction databases, for example, DrugBank database (https://go.drugbank.com/, accessed on 30 January 2022) [82], Therapeutic Target Database (TTD) (http://db.idrblab.net/ttd/, accessed on 30 January 2022) [83], Comparative Toxicogenomics Databases (CTD) (http://ctdbase.org/, accessed on 30 January 2022) [84], and GeneCards (https://www.genecards.org/, accessed on 30 January 2022) [85]. The selected drugs were confirmed the interaction significance using the STITCH v5.0 database (http://stitch.embl.de/, accessed on 30 January 2022) [86], a drug–protein interaction database, by considering a confidence score greater than 0.400 (medium confidence). The confidence score is calculated based on both experimental and computational evidence, similar to the STRING database. Drugs that met the criteria were considered candidate-targeted drugs.

#### 2.8.2. Molecular Docking of Potential Drugs against B-Myb

Molecular docking was performed to elucidate the interaction between drug candidates and a target protein named B-Myb. This protein is encoded from *MYBL2*, an essential gene in the network analysis. The crystal structure of B-Myb was received from PDB (https://www.rcsb.org/, accessed on 12 June 2022) [87] using PDB ID: 6C48 from the study [88]. The function of B-Myb is activated via binding between the LXXLL motif located in the B-Myb transactivation domain and the KIX domain of coactivator p300 to form a transcriptional module [89,90,91,92]. Thus, the motif containing L688, R687, G686, L685, and L684 residues was set as the binding site. Several studies have shown that plumbagin, a natural naphthoquinone binding at this motif, can cause B-Myb/p300 interaction interference [93,94,95]; therefore, plumbagin was used as a reference ligand in the docking study to compare with candidate drugs such as doxorubicin and camptothecin. The three compounds were individually docked into B-Myb using HDOCK server (http://hdock.phys.hust.edu.cn/, accessed on 12 June 2022) [96] and AutoDock VinaXB, a docking program using a genetic algorithm [97]. The ionized states of B-Myb were configured at pH 7.4 using PROPKA3.1 [98], while ChemAxon [99] was used to check the pKa value of the compounds. The binding affinity of the candidate drugs was calculated and compared to plumbagin. The 3D and 2D structures demonstrating the drug–protein interactions were visualized using the UCSF Chimera package [100] and the LigPlot [101].

## 3. Results

### 3.1. Identification of Common DEGs

**Figure 2:**
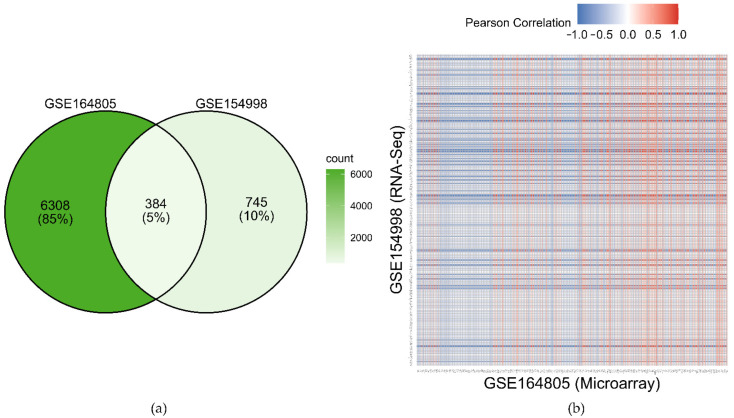
Identifying the common DEGs between the two transcriptomic GEO datasets (GSE164805 and GSE154998). (**a**) Venn diagram of the DEGs found in the datasets. (**b**) Correlation heatmap of the common DEGs between the two datasets.

The common DEGs were filtered from the transcriptomics data based on microarray and RNA-Seq dataset (see Material and Methods) by considering FDR *q*-value < 0.05 and |log2 FC| > 1. There were 6692 and 1129 DEGs found by microarray technique and RNA-Seq technology, respectively. Figure 2a displays the Venn diagram representing the common DEGs from both datasets. In total, 384 common DEGs were identified, having 39 upregulated and 221 downregulated DEGs; however, the remaining common DEGs (124 genes) had both upregulation and downregulation because their expression pattern was contradictory between the two datasets. Figure 2b shows the correlation heatmap of the common DEGs between the two datasets. The gene list of the common DEGs is shown in Table S1 in Supplementary Materials.

### 3.2. Functional Enrichment Analysis of Up- and Downregulated DEGs

**Figure 3:**
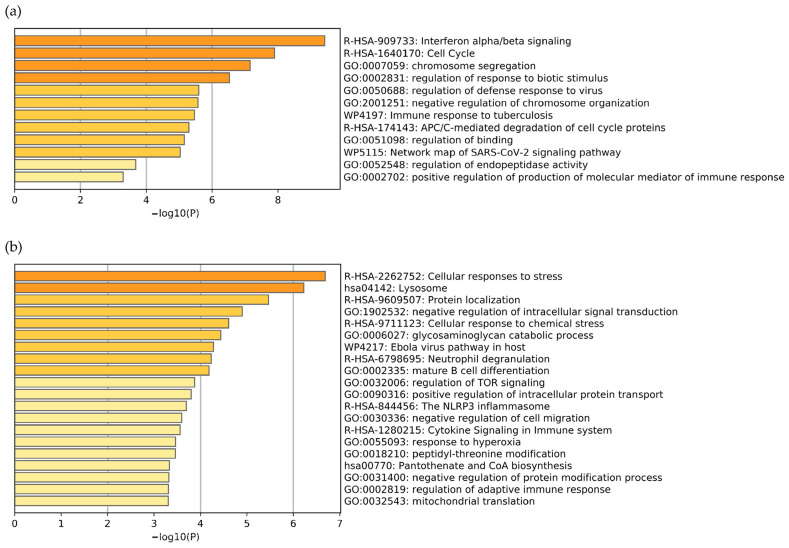
The bar graph represents the enrichment terms analyzed from (**a**) the upregulated DEGs and (**b**) the downregulated DEGs at a significant level (FDR < 0.01). Each enrichment term is colored based on the significance level.

The functional enrichment analysis using Metascape of the DEGs is shown in Figure 3. In the upregulated DEGs, the terms were primarily enriched relevantly to viral innate immune response and cell cycle regulation (Figure 3a). For instance, IFN-α and IFN-β were type I IFN (IFN-I) predominant in the viral innate immune response. In addition, anaphase-promoting complex/cyclosome (APC/C), a cell cycle regulator complex, and chromosome segregation were enhanced in leukocytes during severe COVID-19. Other increased functional terms, such as regulation of binding and endopeptidase activity, were also found in the upregulated DEGs. Moreover, the functional enrichment in the downregulated DEGs was mainly associated with cellular response to stress, lysosome, protein localization (the processes establishing and maintaining proteins at specific locations), glycosaminoglycan catabolic pathway, mature lymphocyte differentiation, positive regulation of intracellular protein transportation, negative regulation of protein modification, response to hyperoxia, and adaptive immune response (Figure 3b).

### 3.3. PPI Network Construction, Topological Analysis, and Cluster Detection

From the PPI network construction without the neighboring node expansion via STRING v11.0, there were 85 components with 384 nodes and 861 edges. The largest component containing 288 nodes and 848 edges was extracted for topological analysis and identifying clusters and key genes. The edge list information for the component is also provided in Table S2 in Supplementary Materials. Global topological parameters calculated from the Network Analyzer plugin in Cytoscape are illustrated in Table 1. Moreover, local topological parameters in each node in the network are summarized in Table S3 in Supplementary Materials.

**Figure 4:**
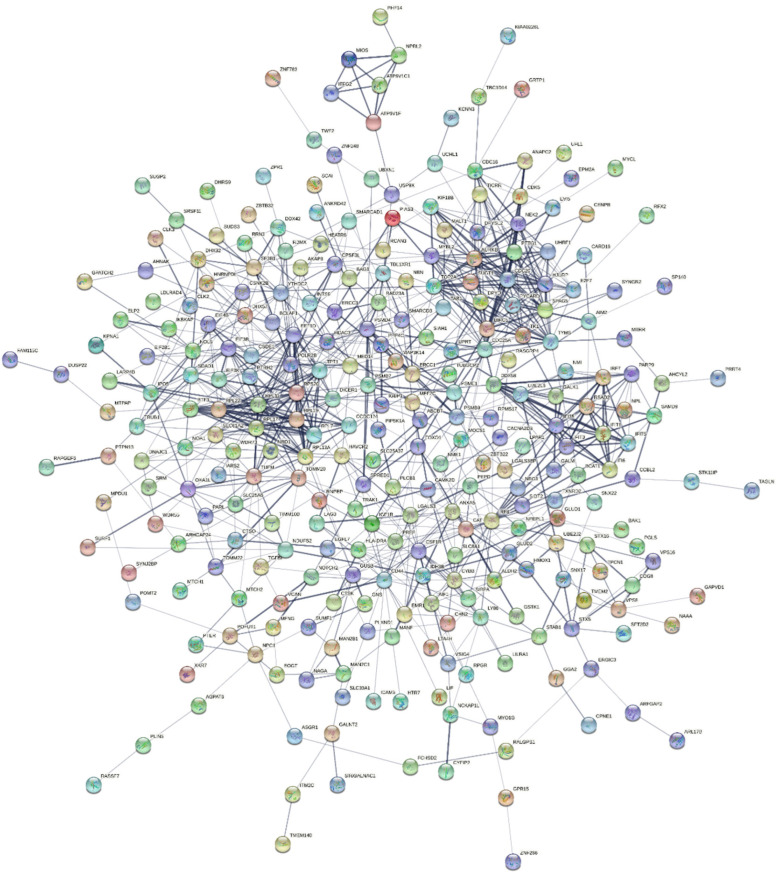
The largest component of the PPI network constructed from the common DEGs visualized by STRING v11.0 with the interaction confidence score > 0.400 (medium confidence). The network consists of 288 nodes and 848 interactions.

The largest network visualized by STRING v11.0 is shown in Figure 4. The results analyzed by the STRING revealed that the average node degree, expected number of edges, and average local clustering coefficient were 5.89, 544, and 0.461, respectively. Additionally, a PPI enrichment *p*-value was less than 10^−16^, indicating that the proteins have interactions with each other more than by chance.

**Figure 5:**
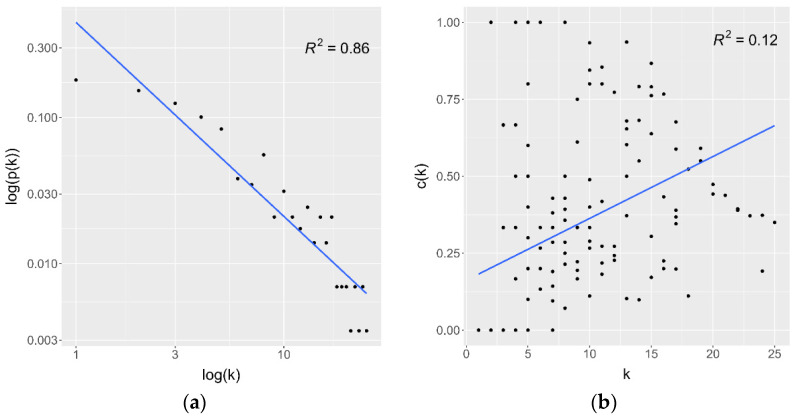
Topological analysis of the PPI network. (**a**) Degree distribution plot. (**b**) Clustering coefficient versus degree plot. *k* denotes the degree; *p*(*k*) denotes the probability of degree *k*; *c*(*k*) denotes the clustering coefficient of a node that has degree *k*.

The network probably provided the small-world effect, such as several biological networks, because it had a low value of the mean shortest path length (*mspl* = 4.33) even though there was a moderate average clustering coefficient (*acc* = 0.28). Furthermore, the degree distribution plot illustrated in Figure 5a shows the power-law property, indicating the strong negative association between logarithmic scales of degree and its probability (*R^2^* = 0.86). On the other hand, the clustering coefficient versus degree plot (Figure 5b) shows no relationship between the clustering coefficient and degree (*R^2^* = 0.12). These behaviors suggested that the network had scale-free properties.

**Figure 6:**
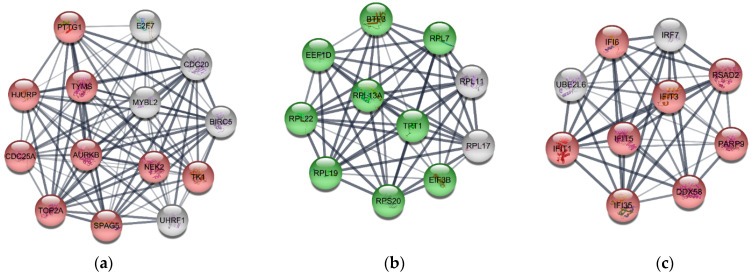
Cluster detection of the PPI network using MCODE plugin in Cytoscape 3.9.0. (**a**) MCODE 1 had 14 nodes and 89 edges. (**b**) MCODE 2 had 11 nodes and 53 edges. (**c**) MCODE 3 had 10 nodes and 45 edges. The red and green nodes represent upregulated and downregulated DEGs. In contrast, gray nodes represent genes having both upregulation and downregulation.

There were three clusters identified from the MCODE plugin with the score of more than 5: MCODE 1, 2, and 3. Topological parameters of the clusters were described in Supplementary Table S4. Most MCODE 1 and 3 cluster members were upregulated DEGs, while MCODE2’s cluster members were downregulated DEGs. Functional enrichment results of each cluster are illustrated in Table 2 and Supplementary Figure S1. For instance, MCODE 1 (Figure 6a) is enriched in the cell cycle and division regulation process, while MCODE 2 (Figure 6b) is concentrated in the translation process and transactivation response element RNA-binding protein (TRBP). Moreover, MCODE 3 (Figure 6c) is associated with an innate immune response.

### 3.4. Finding Potential miRNAs as Novel Biomarkers in Regulatory Networks

**Figure 7:**
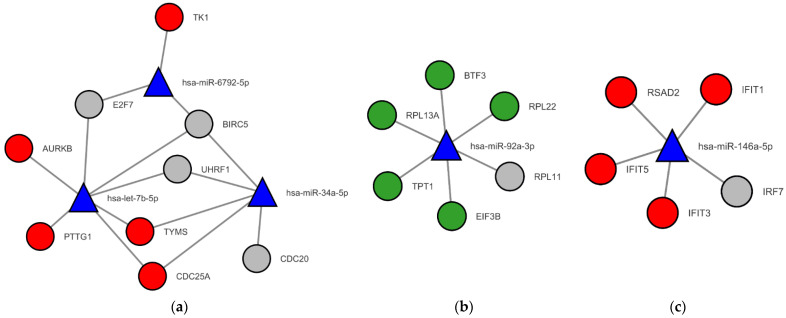
miRNA–mRNA interaction regulatory networks based on MCODE clusters from the PPI network. The networks were bipartite graphs. The regulatory network of (**a**) MCODE 1 had 12 nodes and 15 edges, (**b**) MCODE 2 had 7 nodes and 6 edges, and (**c**) MCODE 3 had 6 nodes and 5 edges. The blue triangular nodes are miRNAs. The red and green circular nodes represent upregulated and downregulated DEGs, respectively. In comparison, gray nodes represent genes having both upregulation and downregulation.

Figure 7 illustrates miRNA–mRNA interaction networks constructed based on the three MCODE clusters. The interactions were statistically significant at FDR *q*-value < 0.05. There were five novel candidate biomarkers analyzed from the regulatory networks, for instance, hsa-miR-6792-5p, hsa-let-7b-5p, hsa-miR-34a-5p, hsa-miR-92a-3p, and hsa-miR-146a-5p. The further statistical and interaction data of regulatory networks in MCODE 1, 2, and 3 are explained in Figure S2 and Tables S5–S7 in Supplementary Materials. There were three miRNAs interacting with the mRNAs in MCODE 1 (Figure 7a): hsa-miR-6792-5p, hsa-let-7b-5p, and hsa-miR-34a-5p. In addition, hsa-miR-92a-3p and hsa-miR-146a-5p interacted with the mRNAs in MCODE 2 and 3, respectively. miRNA regulates gene expression via mRNA binding and increases mRNA degradation or activation [102,103]. A change in miRNA levels can indicate gene expression status; therefore, miRNA measurement can be applied to predict severe COVID-19 based on the effect on gene expression patterns.

### 3.5. Key Gene Identification and Survival Analysis

There were 19 and 15 genes being hub and bottleneck, respectively. Tables S8 and S9 in Supplementary Materials reveal topological parameters of the hub and bottleneck genes, such as degree, betweenness, closeness, and clustering coefficient. Furthermore, Figure S3 in Supplementary Materials shows a Venn diagram of nodes being the hub and bottleneck genes. Seven genes were both hub and bottleneck: *AURKB*, *CD44*, *CDC25A*, *DDX58*, *DICER1*, *POLR2B*, and *RPL7*. Table 3 displays the biological function of the hub and bottle genes. Most hub genes were involved in cell proliferation and differentiation, such as cell cycle regulation, hematopoiesis, antiapoptotic process, DNA replication and transcription, and ribosomal synthesis. Additionally, the bottleneck genes in the PPI network mainly play an essential role in inflammation, antiviral and innate immune activation, oxidative stress prevention, and biomolecule metabolisms, for instance, lymphocyte and macrophage activation, viral recognition, mitochondrial protein transportation, protein and glycosaminoglycan degradation, and heme catabolism.

**Figure 8:**
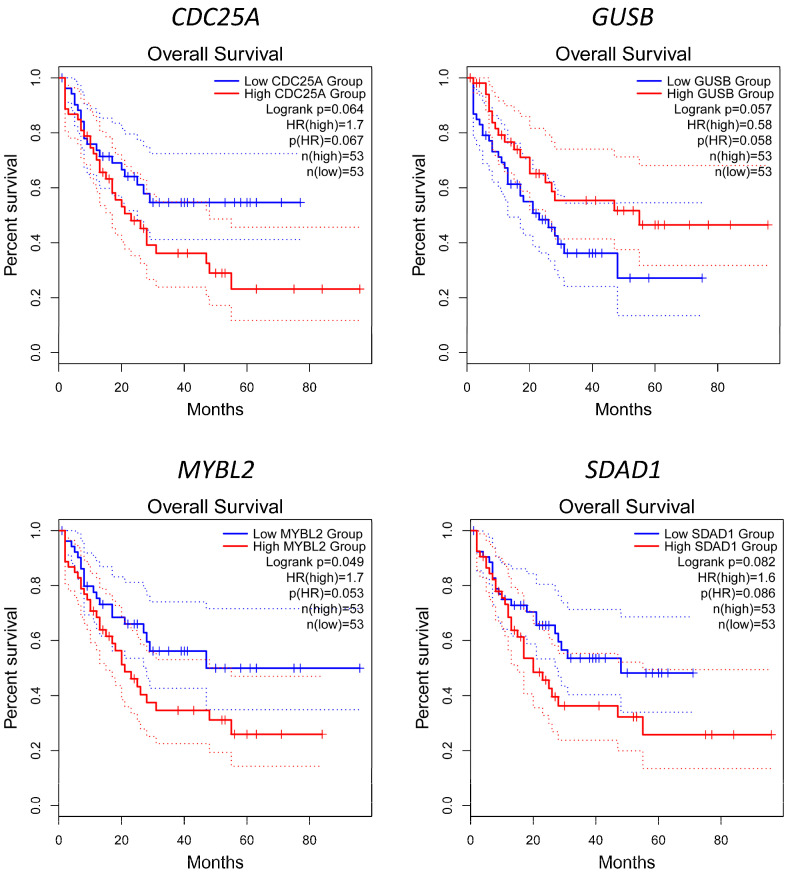
Kaplan–Meier overall survival analysis of the hub and bottleneck genes with significant or almost significant log-rank *p*-value: *CDC25A*, *GUSB*, *MYBL2*, and *SDAD1*. The curves were plotted using Gene Expression Profiling Interactive Analysis (GEPIA2). Acute myeloid leukemia (LAML) from The Cancer Genome Atlas (TCGA) database was used as a cell type model to find key survival genes in cytokine storm-related myeloid cells such as neutrophils, monocytes, and macrophages.

The survival analysis using GEPIA2 based on the LAML model in the TCGA database of the 27 hub and bottleneck genes revealed that only *MYBL2* provided significant overall survival (log-rank *p*-value < 0.05) and a high hazard ratio (HR = 1.7); however, there were three genes that were nearly significant overall survival and high hazard ratio, for example, *CDC25A* (log-rank *p*-value = 0.064 and HR = 1.7), *GUSB* (log-rank *p*-value = 0.057 and HR = 0.58), and *SDAD1* (log-rank *p*-value = 0.082 and HR = 1.6). Figure 8 displays Kaplan–Meier overall survival analysis of the significant and almost significant genes. The overall survival analysis of other hub and bottleneck genes is illustrated in Figure S4 in Supplementary and Materials.

### 3.6. Finding Candidate Targeted Drugs

**Figure 9:**
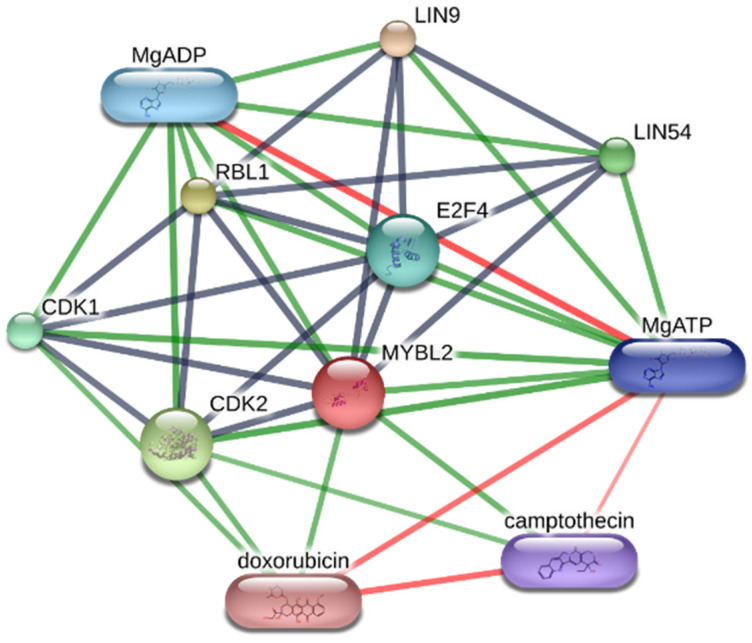
Drug–protein interaction network of the candidate drugs targeting *MYBL2* resulted from STITCH v5.0. The black, green, and red edges represent protein–protein, drug–protein, and drug–drug interactions.

*MYBL2*, the significant key gene obtained from the survival analysis, was inputted to the drug–gene interaction databases: DrugBank database [82], TTD [83], CTD [84], and GeneCards [85]. The almost significant key genes, such as *CDC25A*, *GUSB*, and *SDAD1*, were also used to find drug–gene interactions. The result showed 35 FDA-approved drugs interacting with the key genes, as illustrated in Table S10 in Supplementary Materials. STITCH v5.0 database [86] was used to confirm the result from the search. *MYBL2* was the only key gene having drug–protein interaction. The STITCH result revealed that doxorubicin and camptothecin interact with *MYBL2*, as shown in Figure 9.

**Figure 10:**
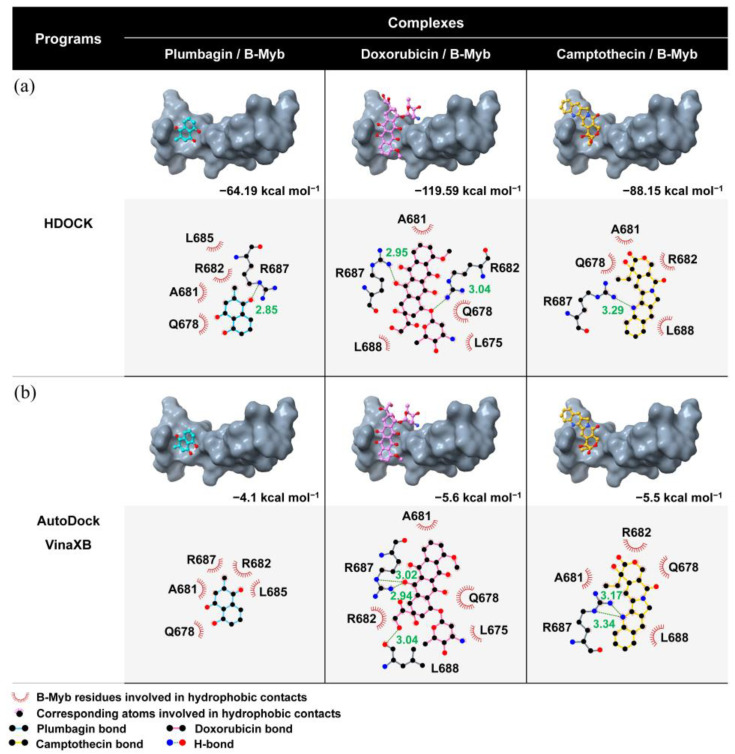
Binding orientation and interaction of the two focused drug candidates with the LXXLL motif of B-Myb compared to plumbagin/B-Myb complex via (**a**) HDOCK webserver and (**b**) AutoDock VinaXB. HDOCK scores and binding affinities of all complexes are also shown.

Figure 10 displays the molecular docking results of the studied compounds binding to the LXXLL motif by the HDOCK webserver (Figure 10a) and AutoDock VinaXB (Figure 10b). The former program showed that either plumbagin or candidate drugs interacted with the three crucial residues associated with the motif, i.e., L685, R687, and L688. Through the interaction with the active site of B-Myb, doxorubicin and camptothecin produced an HDOCK score of −119.59 and −88.15 kcal mol^−1^, relatively outperforming plumbagin’s (−64.19 kcal mol^−1^). The strong binding affinity of doxorubicin was supported by two hydrogen bonds formed with the two positively charged residues, R682 and R687. Conversely, only one hydrogen bond binding to residue R687 was detected in the reference ligand and camptothecin. The obtained data were in accordance with the AutoDock VinaXB results. All compounds can bind to the critical residues L685, R687, and L688 with binding affinities of −4.1, −5.6, and −5.5 kcal mol^−1^ for plumbagin, doxorubicin, and camptothecin, respectively. Again, there were hydrogen bonds between the candidate drugs and B-Myb through R682 and R687 residues. In contrast, no hydrogen bond formation was identified in the case of the reference ligand.

## 4. Discussion

Finding novel biomarkers, key genes, and candidate targeted drugs is necessary to predict, treat, and follow severe COVID-19 patients. This study conducted various types of biological network analysis, such as regulatory and protein–protein interaction networks, based on common DEGs from microarray data and RNA-Seq data for the transcriptomics data of severe CPVID-19 patients. The functional enrichment analysis of the upregulated and downregulated DEGs was operated to discover the disease’s underlying molecular mechanisms. We also detected a network community in the PPI network. Novel biomarkers were discovered via miRNA identification in the regulatory networks constructed based on the MCODE modules. In addition, the key genes in the PPI network were found by finding the hub and bottleneck nodes using degree and betweenness centrality measurement and were validated by the overall survival analysis of the LAML model. Finally, drug repurposing was performed by drug–gene and drug–protein interaction database searching and molecular docking based on the key genes.

We identified 384 common DEGs that met the two datasets, and the number of upregulated and downregulated genes were 39 and 221, respectively. The remaining 124 DEGs had both upregulation and downregulation. The functional enrichment result of the upregulated DEGs revealed that the terms were generally involved in antiviral and innate immune response and cell cycle regulation. The processes and pathways were concordant with immune responses to infectious diseases. In host response to infections, immune-related, inflammatory-related, and leukocyte proliferation and differentiation genes are overexpressed to eradicate pathogens [104,105,106,107]; however, excessive immune and inflammatory responses can cause uncontrolled self-tissue injury, leading to severe complications and increased morbid and mortal cases. Furthermore, the enrichment analysis of the downregulated DEGs mainly concentrated in the cellular response to stress, lysosome, mature lymphocyte differentiation, negative regulation of protein modification, glycosaminoglycan catabolic pathway, response to hyperoxia, and adaptive immune response. Numerous studies have shown that impaired lymphocyte differentiation and adaptive immune activation are found in severe COVID-19, resulting in delayed viral clearance and persistent proinflammatory cytokine release [108,109,110,111,112]. ARDS and severe pneumonia are also found in severe COVID-19, causing hypoxia. Hence, genes related to the hyperoxia response were downregulated. In addition, negative protein modification regulation expression was reduced to increase the proinflammatory cytokine and antiviral protein production and release. Decreased glycosaminoglycan degradation can promote SAR-CoV, MERS-CoV, and SARS-CoV-2 to infect host cells. For example, some studies have revealed that the viruses use S protein binding with heparan sulfate proteoglycans (HSPGs) to enter the host cells in the disease’s early stage [113,114,115,116].

The PPI network constructed by the STRING database based on the common DEGs, same as other biological networks, had the scale-free property. The scale-free property was proved by the strong relationship between degree and degree probability in degree distribution and the independence of the clustering coefficient and degree. Furthermore, the network likely provided the small-world effect because it had a low average shortest path length and moderate average clustering coefficient. The PPI network cluster detection using the MCODE algorithm showed three clusters with high MCODE scores: MCODE 1, 2, and 3. MCODE 1 was the upregulated gene cluster mainly enriched in cell proliferation and cell cycle. MCODE 2, the downregulated gene set, was primarily concentrated in ribosomal synthesis and protein translation regulation. Furthermore, MCODE 3 was centered on antiviral and innate immune responses; therefore, the enrichment terms of each cluster were according to the terms found in upregulated and downregulated DEGs.

The regulatory networks from the MCODE clusters showed that five miRNAs, hsa-miR-6792-5p, hsa-let-7b-5p, hsa-miR-34a-5p, hsa-miR-92a-3p, and hsa-miR-146a-5p, were the novel candidate biomarkers. hsa-miR-6792-5p, hsa-let-7b-5p, and hsa-miR-34a-5p interacted upregulated mRNAs related to cell proliferation and differentiation in MCODE 1 cluster. In MCODE 2, downregulated mRNAs involved in protein translation regulation were associated with hsa-miR-92a-3p. Moreover, hsa-miR-146a-5p interacted with upregulated antiviral and innate immune mRNAs in MCODE 3. miRNAs are small, non-coding RNAs that play an essential role in controlling gene expression via binding mRNA and then increase mRNA cleavage or translation dependent on their properties [103,117]. miRNAs also have a role in clinical applications such as diagnostic markers and therapeutic targets [118,119]. Because miRNAs are stable and detectable in serum and plasma, they are applied as biomarkers for diagnosis [120]. Several studies have revealed miRNA expression based on viral proteins, host membrane receptors, and proinflammatory cytokines [121,122,123,124]; however, no study has reported the relationship between the five miRNAs acquired from the regulatory network analysis and COVID-19. Thus, they could play a crucial role in novel diagnostic biomarkers and therapeutic agents in severe COVID-19 and further investigation of their roles should be needed.

There were 27 hub or bottleneck genes from high degree and betweenness value selection. The hub genes were mainly involved in cell proliferation and differentiation, while the bottleneck genes were focused on antiviral and innate immune responses. We also found the four key genes, such as *CDC25A*, *GUSB*, *MYBL2*, and *SDAD1*, from the overall survival analysis based on the LAML model. *CDC25A* is a cell cycle and apoptosis regulator that plays a vital role in many cancers’ progression, for example, breast, esophageal, lung, colorectal, prostate, and ovarian cancer [125,126]. Furthermore, in viral infection, a study performing Sendai virus-infected cell line showed that upregulated *CDC25A* suppressed IFN-β activation while knockdown of *CDC25A* increased IFN-β stimulation [127]. The result suggested that *CDC25A* could participate in impaired viral innate immunity and increase viral survival. *GUSB* is a hydrolase enzyme for glycosaminoglycan degradation [128]. As described earlier, declined glycosaminoglycan degradation can promote the coronaviruses to enter the host cells. As a result, *GUSB* can play a central role in COVID-19 progression. B-Myb, encoded from *MYBL2*, is a transcription factor in the MYB family that plays an essential role in cell proliferation, differentiation, apoptosis, and tumorigenesis [129]. It is used as a prognostic marker in many cancer types, such as hepatocellular carcinoma, gallbladder, colorectal, and breast cancer [130,131,132,133]. Interestingly, a weighted gene co-expression network analysis in the COVID-19 study reported that *MYBL2* was one of 52 hub genes from the network analysis [134]. This result suggested the important role of *MYBL2* in numerous biological networks. There are a few studies on the role of *SDAD1*. It probably plays a role in ribosomal biogenesis and tumorigenesis [135]. There is no report about a relationship between its expression and COVID-19. Hence, further studies on the biological roles of *SDAD1* are needed.

The candidate targeted drug discovery came from searching in the four drug–gene interaction databases and the drug–protein interaction database based on the four key genes. The result indicated that doxorubicin and camptothecin had interacted with *MYBL2*. The drug–protein interactions can be investigated by molecular docking. No 3D structure of B-Myb in complex with known inhibitor is currently available. The involvement between the key residues and binding site in B-Myb’s LXXLL motif, a multifunctional binding sequence in transcriptional regulation [136], was reported [93,94,95]. The b-Myb activity was inhibited by blocking the KIX domain of the B-Myb interaction partner, which was p300 [89], through natural [137,138,139] and small compounds [140]; however, identifying compounds that inhibit directly on B-Myb rather than p300 has not been revealed. In this work, the molecular docking results from the HDOCK webserver and AutoDock VinaXB showed that the two drug candidates, doxorubicin and camptothecin, had physical interactions with B-Myb. This evidence was supported by a reduction in cell proliferation in cancer cell lines having *MYBL2* overexpression without proving apparent mechanisms [141,142]. We then proposed the possible mechanism from our study that their direct interactions with B-Myb could be involved in the decreased cellular activity of upregulated *MYBL2* cells. Additionally, the candidate drugs demonstrated binding interaction and susceptibility with B-Myb significantly greater than plumbagin, the reference ligand; therefore, doxorubicin and camptothecin could be potential candidates to combat COVID-19.

There is other evidence to support that the candidate drugs could play an important role in severe COVID-19 treatment. Doxorubicin is a chemotherapeutic agent treating various types of cancer [143]. A study of structural bioinformatics revealed that doxorubicin proved the significant binding energy with SARS-CoV-2 main protease in the molecular docking [144]. This result suggested that doxorubicin could be a potential drug to treat severe COVID-19. Camptothecin is a natural product extracted from the Chinese happy tree (*Camptotheca acuminata*) [145]. It is used as a chemotherapeutic agent in cancer treatment by inhibiting DNA replication [146,147]. Camptothecin also has antiviral activity by inhibiting viral replication [148,149,150]. A study on transcriptomic profile in COVID-19 using bioinformatics showed that camptothecin could reverse the gene signature in COVID-19 [151]. In addition, the evidence from a molecular docking study uncovered that camptothecin formed hydrogen bonds with SARS-CoV-2 S protein to prevent the binding between S protein and ACE2 receptor [152]. The results indicated that camptothecin could play a vital role in COVID-19 treatment.

We studied the biological networks and structural biology to identify the key genes, novel biomarkers, and candidate targeted drugs based on leukocyte transcriptomic profiles; however, the immunopathology of severe COVID-19 is the interaction between immune cells and respiratory cells. Analysis of peripheral white blood cell gene expression can lose some proinflammatory cytokine information. Performing lung transcriptomic profiles for biological network construction is our suggestion for future research. Single-cell methods should be conducted to identify key genes and targeted drugs in each cell type. Advanced computational chemical methods such as molecular mechanics and molecular dynamics should also be included to simulate drug–protein interactions. Moreover, machine learning approaches are needed to deal with the big data of transcriptomic profiles to find the important features and predict key genes, novel biomarkers, and candidate-targeted drugs more widely and precisely.

## 5. Conclusions

Our study performed the multi-level biological network analysis from peripheral white blood cell transcriptomic profiles in severe COVID-19 patients. We found that the upregulated genes were enriched in cell proliferation and innate immune responses while the downregulated genes were concentrated in lymphocyte differentiation, adaptive immune response, and glycosaminoglycan degradation. The regulatory network analysis of the PPI network clusters provided novel diagnostic biomarkers from miRNAs. The key genes in severe COVID-19 were also identified via topological and survival analysis. These key genes play a significant role in leukocyte proliferation, antiviral activity, and viral proliferation. Furthermore, the candidate drugs targeting the key genes were found from database searching and evaluated with molecular docking. Nonetheless, other biomarkers, key genes, and candidate-targeted drugs were not found and need further investigation; therefore, advanced experimental and computational tools should be integrated to find new biomarkers and target treatments more precisely and personally.

## Figures and Tables

**Table 1:** Global topological parameters of the PPI network.

Symbol	Description	Value
*N*	Number of nodes	288
*M*	Number of edges	848
<*k*>	Average degree	5.89
*d*	Diameter	11
*r*	Radius	7
*mspl*	Mean shortest path length	4.33
*D*	Density	0.02
*acc*	Average clustering coefficient	0.28

**Table 2:** Functional enrichment analysis of the MCODE clusters using Metascape.

Cluster	Cluster Score	Term ID	Biological Term	Count	Log10 (*q*-Value)
MCODE1		R-HSA-1640170	Cell cycle	11	−11.20
	13.692	GO:0007059	Chromosome segregation	7	−6.85
		M40	PID E2F pathway	5	−6.28
		GO:1903047	Mitotic cell cycle process	7	−5.60
MCODE2	10.600	R-HSA-156842	Eukaryotic translation elongation	8	−13.67
		R-HSA-72766	Translation	9	−12.97
		CORUM:5380	TRBP containing complex (DICER, RPL7A, EIF6, MOV10, and subunits of the 60S ribosomal particle)	3	−4.38
MCODE3	10.000	R-HSA-913531	Interferon signaling	9	−14.37
		GO:0051607	Defense response to virus	8	−11.52
		WP4197	Immune response to tuberculosis	3	−3.91
		GO:0002831	Regulation of response to biotic stimulus	3	−3.91

**Table 3:** Summary of the biological functions of 27 genes which were hub or bottleneck.

Symbol	Description	Node Property	Biological Function
*ANXA5*	Annexin A5	bottleneck	Inflammation, growth, and differentiation
*AURKB*	Aurora Kinase B	hub, bottleneck	Cell cycle regulation
*BIRC5*	Baculoviral IAP Repeat Containing 5	hub	Antiapoptotis
*CAT*	Catalase	bottleneck	Oxidative stress prevention
*CD44*	Cluster of Differentiation 44	hub, bottleneck	Hematopoiesis and lymphocyte activation
*CDC20*	Cell Division Cycle 20	hub	Cell cycle regulation
*CDC25A*	Cell Division Cycle 25A	hub, bottleneck	Cell cycle regulation
*CSF1R*	Colony Stimulating Factor 1 Receptor	bottleneck	Macrophage differentiation
*DDX58*	DExD/H-Box Helicase 58	hub, bottleneck	Viral dsRNA recognition
*DICER1*	Ribonuclease III	hub, bottleneck	Small RNA production and antiviral agent
*EEF1D*	Eukaryotic Translation Elongation Factor 1 Delta	hub	Transport tRNAs to ribosome
*GUSB*	Glucuronidase Beta	bottleneck	Glycosaminoglycan degradation
*HMOX1*	Heme Oxygenase 1	bottleneck	Heme catabolism
*MYBL2*	MYB Proto-Oncogene Like 2	hub	Cell cycle regulation
*POLR2B*	RNA Polymerase II Subunit B	hub, bottleneck	DNA transcription
*PSMD4*	Proteasome 26S Subunit Ubiquitin Receptor, Non-ATPase 4	bottleneck	Protein degradation
*RPL7*	Ribosomal Protein L7	hub, bottleneck	A protein component in ribosomes
*RPL11*	Ribosomal Protein L11	hub	A protein component in ribosomes
*RPL13A*	Ribosomal Protein L13a	hub	A protein component in ribosomes
*RPL17*	Ribosomal Protein L17	hub	A protein component in ribosomes
*RPL19*	Ribosomal Protein L19	hub	A protein component in ribosomes
*RPS20*	Ribosomal Protein S20	hub	A protein component in ribosomes
*SDAD1*	SDA1 Domain Containing 1	hub	Ribosomal production and transportation
*TOMM20*	Translocase Of Outer Mitochondrial Membrane 20	bottleneck	Mitochondrial protein transportation
*TOP2A*	DNA Topoisomerase II Alpha	hub	DNA replication and transcription
*TYMS*	Thymidylate Synthetase	hub	DNA replication and repair
*USP9X*	Ubiquitin Specific Peptidase 9 X-Linked	bottleneck	Similar to ubiquitin-specific proteases

IAP, inhibitor of apoptosis protein; dsRNA, double-strand RNA; tRNA; MYB, myeloblastosis; SDA1, severe depolymerization of actin protein 1.

## Data Availability

The data generated in this study is available in this article.

## Supplementary Materials

The following supporting information can be found and downloaded at: https://www.mdpi.com/article/10.3390/jpm12071030/s1. Table S1: Common differentially expressed genes (DEGs) from the two GEO datasets; Table S2: Edgelist of the largest PPI network; Table S3: Local topological parameters in each node in the PPI network; Table S4: Local topological parameters of the three MCODE clusters; Table S5: Statistical and interaction data of regulatory networks in MCODE 1; Table S6: Statistical and interaction data of regulatory networks in MCODE 2; Table S7: Statistical and interaction data of regulatory networks in MCODE 3; Table S8: Topological parameters of the 19 hub genes in the PPI network; Table S9: Topological parameters of the 15 bottleneck genes in the PPI network; Table S10: Drug-gene interaction data of the key genes; Figure S1: Functional enrichment analysis of genes in each MCODE module; Figure S2: Bar graph of the number of interactions of miRNAs in each gene in MCODE modules using MIENTURNET based on miRTarBase database; Figure S3: Venn diagram of the key genes in the degree and betweenness centrality; Figure S4: Kaplan-Meier overall survival analysis of the hub and bottleneck genes in severe COVID-19.
