# Supplementary material for: Multi-Level Biological Network Analysis and Drug Repurposing Based on Leukocyte Transcriptomics in Severe COVID-19: In Silico Systems Biology to Precision Medicine
Source: J Pers Med. 2022 Jun 23;12(7):1030. doi: 10.3390/jpm12071030 (PMC9319133; doi:10.3390/jpm12071030)
Supplement: Supplementary file 1 [file jpm-12-01030-s001.zip › FigureS.pdf]

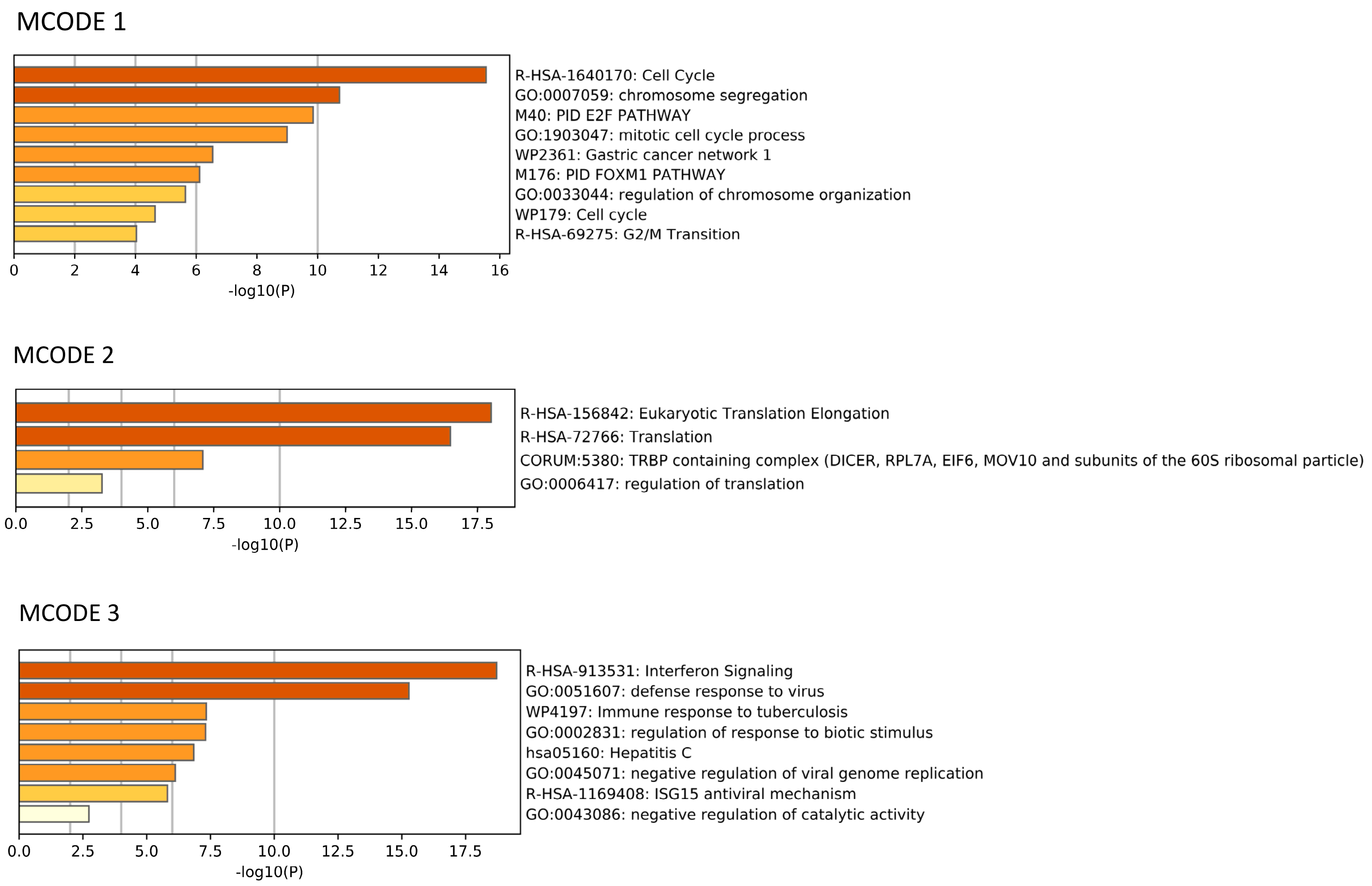

Figure S1. Functional enrichment analysis of genes in each MCODE module. The enrichment graphs were plotted using Metascape.

MCODE 1

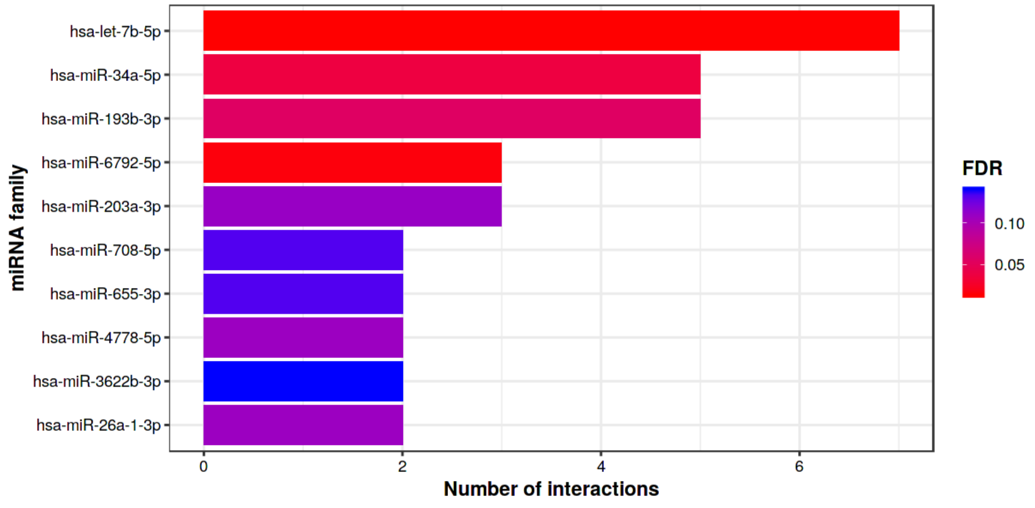

MCODE 2

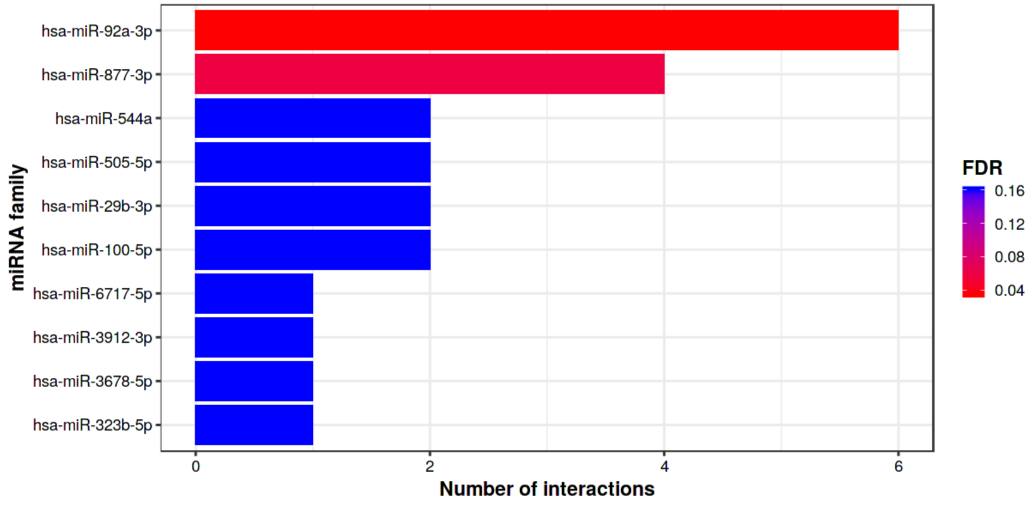

MCODE 3

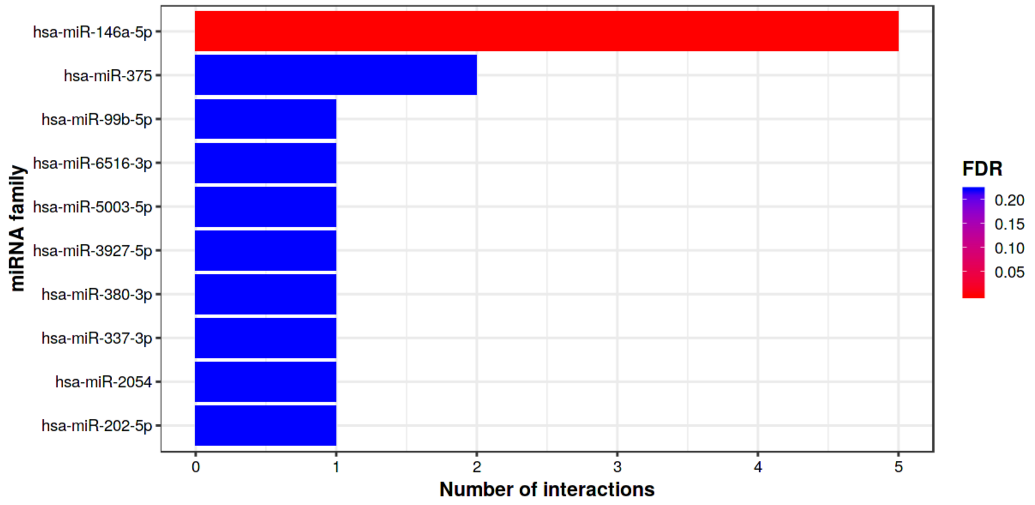

Figure S2. Bar graph of the number of interactions of miRNAs in each gene in MCODE modules using MIENTURNET based on miRTarBase database. The bar graph color represents the interaction significance.

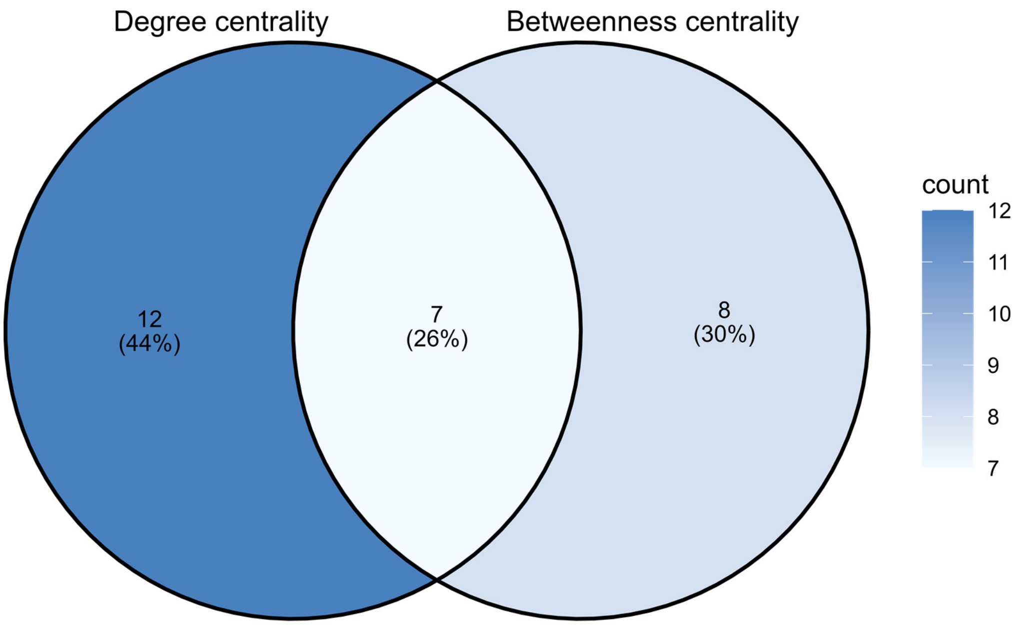

Figure S3. Venn diagram of the key genes in the degree and betweenness centrality.

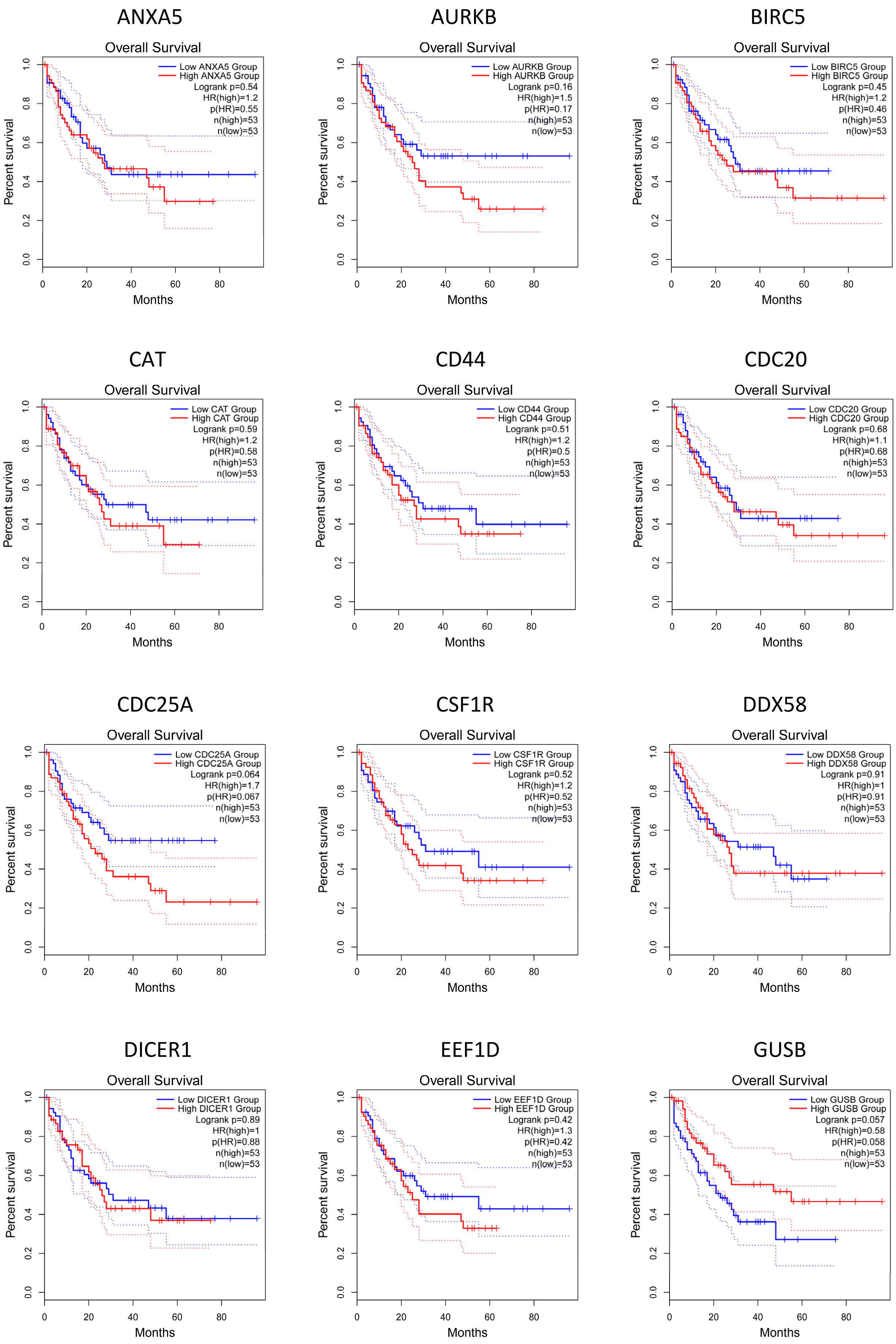

Figure S4. *Cont.*

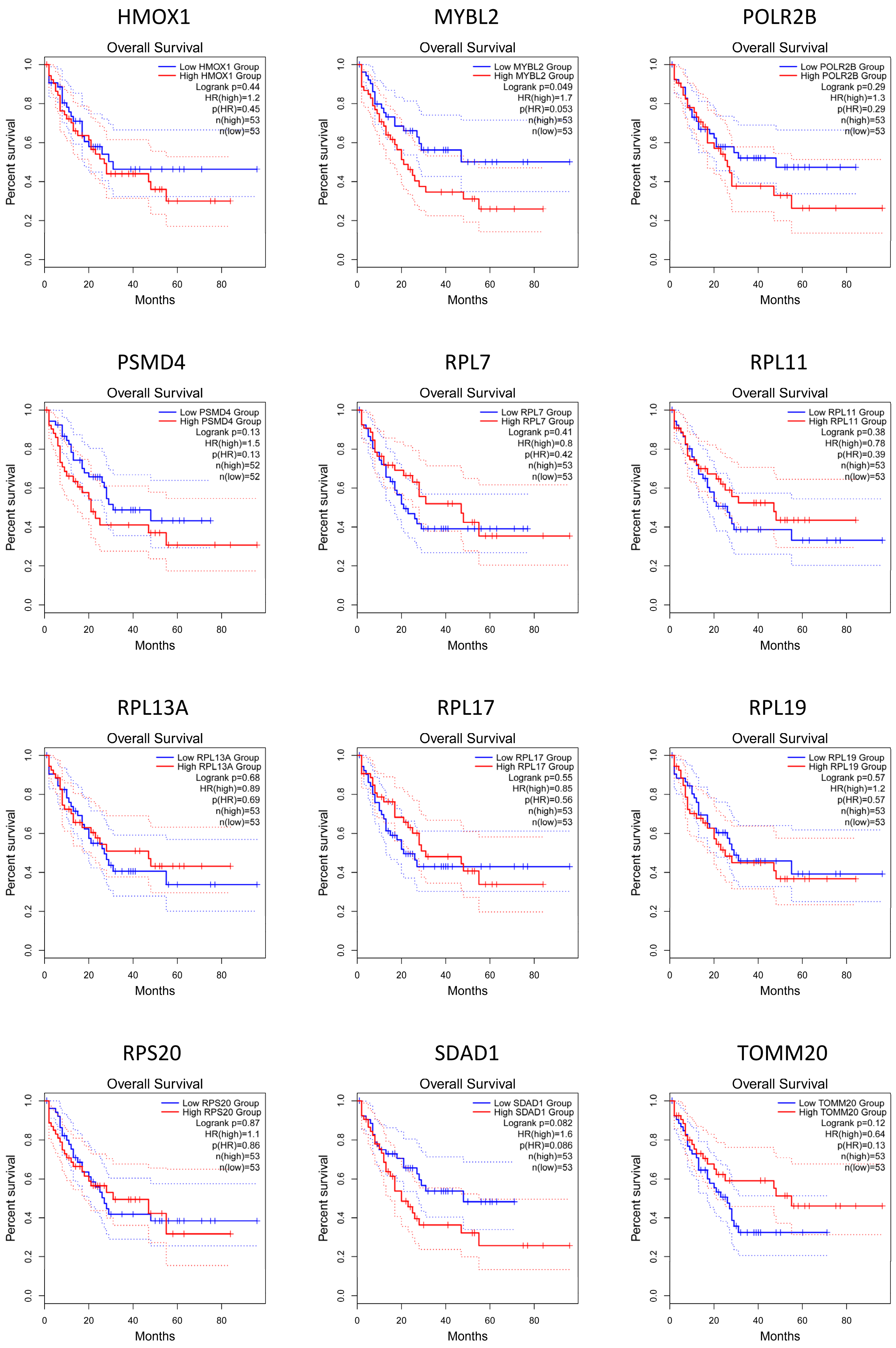

Figure S4. *Cont.*

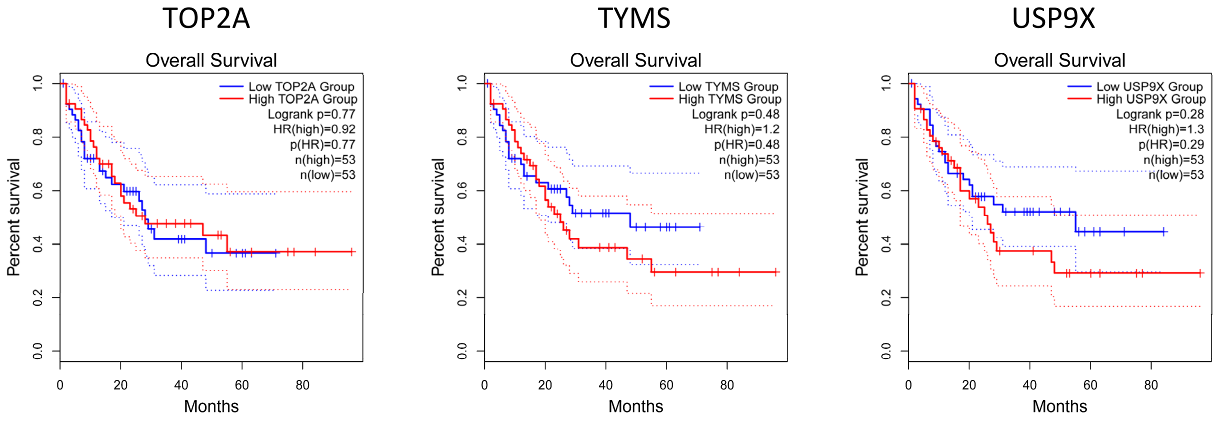

Figure S4. Kaplan-Meier overall survival analysis of the hub and bottleneck genes in severe COVID-19. The curves were plotted using Gene Expression Profiling Interactive Analysis (GEPIA2). Acute myeloid leukemia (LAML) from The Cancer Genome Atlas (TCGA) database was used as a cell type model to find key survival genes in cytokine storm-related myeloid cells such as neutrophils, monocytes, and macrophages.
