# Supplementary material for: Multi-Level Biological Network Analysis and Drug Repurposing Based on Leukocyte Transcriptomics in Severe COVID-19: In Silico Systems Biology to Precision Medicine
Source: J Pers Med. 2022 Jun 23;12(7):1030. doi: 10.3390/jpm12071030 (PMC9319133; doi:10.3390/jpm12071030)
Supplement: Supplementary file 1 [file jpm-12-01030-s001.zip › Table S1.pdf]

Table S1. Common differentially expressed genes (DEGs) from the two GEO datasets *.

DEGs	Gene Symbol
Upregulated	KIF18B, BTN3A1, NEK2, RSAD2, TMEM140, IFI6, SP140, PRRT4, KCNN3, HJURP, TYMS, LEFTY2, ZNF595, ITM2C, ZNF493, PTTG1, CDC25A, SPAG5, NBN, TOP2A, SAMD9, PARP9, IFIT3, AIM2, IFIT1, DDX58, AURKB, CCR10, TICRR, PSMB9, CARD16, GRTP1, MZB1, LGALS3BP, IFI35, PCP2, NMI, IFIT5, TK1
Downregulated	HLA-DRA, EPM2A, NAAA, CD44, MCUB, STARD7, TRIO, IDH3B, KYAT3, RPL13A, EIF4B, MYO1G, HAVCR2, RPL22, RASSF7, ATP6V1F, GLUD1, LPAR1, TAB1, NRG1, CYB561A3, LPXN, RBMX, CSNK2B, DMAC1, SCAI, RPL19, GOLGA8B, EOGT, UBXN1, RAD23A, ADCK5, LGALS3, ALDH2, RALGPS1, CAT, TWF2, PTER, PGLS, LDLRAD4, SDAD1, HDAC3, PTPN13, SUGP2, IRF8, ASB1, MIOS, AHNAK, CTSK, ERGIC3, PSMB7, PYCARD, ZBTB22, MAP3K4, TCAF2, PREP, EID1, NPEPL1, MFNG, CAMK2D, TBC1D9, SLC46A2, XKR7, CREBL2, TSPAN3, RPS20, RASGRP4, MYO9A, TUBGCP2, SRSF11, PLCB1, RRN3, YTHDC2, GLMP, MED14, NCKAP1L, CENPB, ICAM3, POMT2, MAP10, THAP12, GLUD2, MAN2C1, ADGRE1, RUBCNL, IPO5, USP9X, GNS, LY86, ASGR1, CERS5, ASB13, CPNE1, CEMIP2, MPDU1, UFL1, EIF2B1, PEPD, PARL, SNX22, CREBZF, ANXA5, MTCH2, FCN1, RAB34, GPAT4, DDX42, MTPAP, KPNA1, SNX17, NAGA, IARS2, GSTK1, MALT1, GUSB, HMOX1, SF3B1, CCDC124, TXNRD2, OXA1L, DOP1A, KLHL36, WDR55, OPN3, CYFIP2, KIAA1143, NEPRO, DYRK2, GHITM, PTRH2, AKAP8, STK11IP, RTL6, BTF3, ZNF783, EEF1D, ANKRD42, RNPEP, STAB1, DET1, SH3BGRL, SCYL1, EIF3B, TOMM20, COMMD10, UPRT, TGFBI, SURF1, TAGLN, SLC25A6, GIMAP6, BCAT1, CRYBG3, PHF14, ARFGAP2, RPL7, TRUB1, SUMF1, FOXO1, PIP5K1A, CLK2, VPS16, INTS11, NOL6, SLC25A13, MEF2C, NOA1, TOMM22, ZDHHC6, ZPR1, PLXND1, INTS9, SYNJ2BP, PSMD4, SYNGR2, BCLAF1, MRPS17, ERCC3, ZDHHC3, GAPVD1, KDM4C, MYCL, SLC25A37, CSDE1, UBE2J2, SUGT1, ARPIN, DHX57, TUFM, TEPSIN, CTSO, HNRNPDL, MAN2B1, CDC16, TPT1, SMARCAD1, NPC1, ITFG2, GPATCH2, ELP2, ANAPC2, MAP3K14, DUSP22, CDK5, THTPA, WDR73, HEATR6, LRRC58, ARHGAP24, DPYD, GALK1, FLII, NPRL2, LILRA1, NBPF8, LARP4B, MTCH1, PCID2, SMIM7, GGA2, SNTB2
Both up- and downregulated	IGBP1, ZBTB14, ZNF248, DHX32, KIAA0825, BAG1, LTA4H, RPL11, HTR7, GPR15, POFUT1, PLIN5, ABCB7, VSIG4, PPP4C, SLC8A1, DOCK5, IRGQ, CIPC, ST6GALNAC1, CYBB, VPS8, DSC2, PNPLA8, SEC61A2, SPRED1, VCAN, SMARCD3, TBL1XR1, RPL17, FLOT2, RPGR, RFX2, TIMM10B, ATP6V1C1, NOTCH2, STX5, ZNF266, SUDS3, NRDC, WDR91, CHN2, WDR45, KIAA0753, FCHSD2, DICER1, SLC38A6, ZNF785, EIF3K, PIGG, ZDHHC20, BOD1, MDFIC, POLR2B, SLITRK4, MTRR, SIDT2, RCAN3, GDF7, AHCYL2, TBC1D14, FCGRT, COG8, SIAH1, EVI5, SLC39A1, GALNT2, FAM135A, AREL1, FAM168B, ZSWIM6, AIF1, CLK3, ZNF263, SOWAHC, SFT2D2, NPL, IGF1R, WDR37, TPCN1, TRAK1, DPYSL2, PIAS3, CSF1R, ERCC1, ELP1, NDUFS2, SASH1, FAM20A, FAM204A, STX16, SLC22A4, RALGAPA1, CACNA2D3, SPSB3, RAPGEF6, SIRPA, LAG3, INSYN2B, MOCS1, EGFL7, ZBTB32, MANF, BTN3A2, GALM, UHRF1, BAK1, IRF7, SRM, PCDHGA12, DNAJC1, PSME1, E2F7, CDC20, BTN3A3, UBE2L6, DHRS9, UCHL1, ARL17B, KY, BIRC5, MYBL2, LIF, NME1

* Leukocyte transcriptomic profiles came from GSE164805 and GSE154998 datasets. The criterion for being DEGs were FDR q-value < 0.05 and $|\log2\ FC| > 1$. A total of 384 common DEGs were found including 39 upregulated and 221 downregulated DEGs. The remaining 124 genes were found in both up- and downregulated expression.
