# Supplementary material for: Multi-Level Biological Network Analysis and Drug Repurposing Based on Leukocyte Transcriptomics in Severe COVID-19: In Silico Systems Biology to Precision Medicine
Source: J Pers Med. 2022 Jun 23;12(7):1030. doi: 10.3390/jpm12071030 (PMC9319133; doi:10.3390/jpm12071030)
Supplement: Supplementary file 1 [file jpm-12-01030-s001.zip › Table S8-S9.pdf]

Table S8. Topological parameters of the 19 hub genes in the PPIN.

Gene	Degree	Betweenness	Closeness	Clustering coefficient
RPL11	25	0.030191133	0.3157316	0.3500000
POLR2B	24	0.069496043	0.3122960	0.1920290
AURKB	24	0.055793452	0.2983368	0.3731884
RPS20	23	0.035165157	0.3160793	0.3715415
CDC25A	22	0.097609750	0.3261364	0.3896104
RPL13A	22	0.049903596	0.3174779	0.3939394
RPL7	21	0.058813212	0.3265074	0.4380952
BIRC5	20	0.043279916	0.3072805	0.4736842
RPL19	20	0.027497957	0.3143483	0.4421053
CDC20	19	0.014817251	0.2881526	0.5906433
TOP2A	19	0.030785964	0.3037037	0.5497076
CD44	18	0.069909230	0.3017876	0.1111111
TYMS	18	0.039979277	0.2907801	0.5228758
RPL17	17	0.006197219	0.3040254	0.5882353
DICER1	17	0.103047520	0.3295063	0.1985294
MYBL2	17	0.012582020	0.2864271	0.6764706
EEF1D	17	0.025408882	0.3030623	0.3897059
SDAD1	17	0.027197302	0.2864271	0.3455882
DDX58	17	0.082403659	0.3136612	0.3676471

Table S9. Topological parameters of the 15 bottleneck genes in the PPIN.

Gene	Degree	Betweenness	Closeness	Clustering coefficient
CAT	14	0.10530827	0.3076099	0.0989011
DICER1	17	0.10304752	0.3295063	0.1985294
ANXA5	15	0.09965245	0.3265074	0.1714286
CDC25A	22	0.09760975	0.3261364	0.3896104
CSF1R	16	0.08715357	0.3153846	0.2000000
DDX58	17	0.08240366	0.3136612	0.3676471
CD44	18	0.06990923	0.3017876	0.1111111
POLR2B	24	0.06949604	0.3122960	0.1920290
USP9X	7	0.05970566	0.2630614	0.0000000
RPL7	21	0.05881321	0.3265074	0.4380952
GUSB	9	0.05786831	0.2878636	0.1666667
AURKB	24	0.05579345	0.2983368	0.3731884
PSMD4	16	0.05543467	0.3112798	0.2250000
HMOX1	7	0.05278211	0.2847222	0.2857143
TOMM20	13	0.05029161	0.2901921	0.1025641
